# 4D piezoceramic-integrated scaffolds with bioelectric cues for skeletal muscle regeneration

**DOI:** 10.1016/j.bioactmat.2026.06.027

**Published:** 2026-06-24

**Authors:** Noemi Ravaglia, Arianna Rossi, Maurizio Vignolo, Pietro Galizia, Diana Pacheco, Federica Arienti, Carlo Baldisserri, Caitlin M. Guzzo, Monica Montesi, Rosa Mancinelli, Massimiliano Labardi, Tatiana M.F. Patrício, Julia Glaum, Carla Cunha, Elisa Mercadelli, Giorgio Luciano, Silvia Panseri

**Affiliations:** a Institute of Science, Technology and Sustainability for Ceramics (ISSMC), National Research Council of Italy, Via Granarolo 64, Faenza, 48018, Italy; b Department of Neuroscience, Imaging and Clinical Science, University of Studies “G. D'Annunzio”, Chieti, 66100, Italy; c Institute of Chemical Sciences and Technologies “Giulio Natta” (SCITEC), National Research Council of Italy, Via De Marini 6, Genova, 16149, Italy; d Centre for Rapid and Sustainable Product Development (CDRSP), Polytechnic Institute of Leiria, Marinha Grande, 2430-028, Portugal; e Coimbra Chemistry Centre-Institute of Molecular Sciences (CQC-IMS), Department of Chemistry, University of Coimbra, Coimbra, 3004-535, Portugal; f Department of Pharmacy and Biotechnology, University of Bologna, Via Irnerio 48, Bologna, 40126, Italy; g Department of Materials Science and Engineering, Faculty of Natural Sciences, Norwegian University of Science and Technology, Trondheim, Trøndelag, Norway; h Institute for Chemical-Physical Processes, National Research Council of Italy (CNR-IPCF), Largo Pontecorvo 3, Pisa, 56127, Italy; i SeaPower - Association for the Development of the Sea Economy Industrial Park of Figueira da Foz, R. Acácias 40, Figueira da Foz, A 3090 - 380, Portugal; j i3S - Instituto de Investigação e Inovação em Saúde, Universidade do Porto, Rua Alfredo Allen 208, Porto, 4200-135, Portugal

**Keywords:** Piezoelectric ceramics, Ultrasound, Egg white proteins, Stimulus-responsive biomaterials, Volumetric muscle loss, Regenerative medicine

## Abstract

Volumetric muscle loss (VML) poses a significant challenge due to the limited regenerative capacity of skeletal muscle. In this context, emerging four-dimensional (4D) regenerative strategies, which couple biomimetic scaffolds with external dynamic stimuli, have gained increasing attention for their potential to enhance repair outcomes. A novel piezoelectric scaffold based on egg white proteins (EWP) integrated with barium titanate (BTO) particles was developed using a rapid and versatile microwave-assisted method. The optimized EWP:BTO 1:1 scaffolds combine favourable porosity, mechanical compliance, and controlled degradation, closely mimicking native muscle tissue. Corona poling imparts permanent polarisation to the BTO phase, enabling localised electromechanical stimulation under ultrasound. C2C12 myoblasts cultured on these scaffolds exhibited enhanced adhesion, proliferation, infiltration and differentiation. Ultrasound stimulation synergized with polarisation to upregulate early mechanosensitive and electroactive genes. These findings highlight the capacity of piezoelectric scaffolds to integrate structural and dynamic cues, an essential feature of 4D scaffold-based approaches, promoting early myogenic signalling.

A pilot *in vivo* study confirmed biocompatibility and structural integrity over 28 days, with no evidence of local or systemic toxicity, supporting the suitability of the scaffold for further evaluation in VML models. Overall, EWP:BTO 1:1 piezoelectric scaffolds constitute a sustainable, biocompatible, and functionally active 4D platform that converts mechanical energy into targeted electrical cues. By coupling biomimetic architecture with ultrasound-driven electromechanical activation, this approach provides a versatile and non-invasive strategy to modulate muscle regeneration and represents a promising platform for future functional muscle regeneration studies in VML.

## Highlights

• Rapid microwave-assisted EWP:BTO 1:1 scaffold mimics native muscle features.
• Corona poling enables ultrasound-driven electromechanical stimulation.
• Scaffold supports myoblast adhesion, proliferation, infiltration and differentiation.
• Ultrasound synergises with polarisation to boost mechanosensitive and electroactive pathways.
• *In vivo* study confirms biocompatibility and structural stability.

## Graphical abstract

**Figure:**
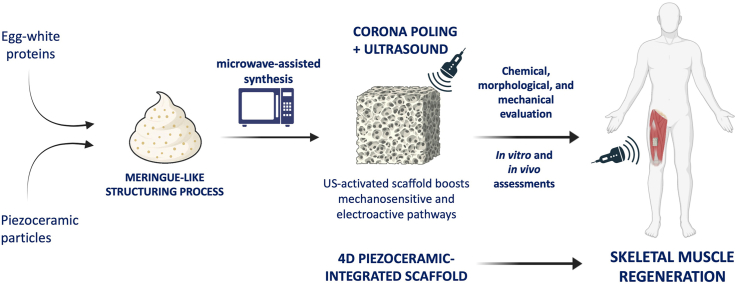

## 1 Introduction

Volumetric muscle loss (VML) represents a critical clinical challenge due to the insufficient regenerative capacity of skeletal muscle to recover from extensive tissue loss. VML involves the irreversible damage of a large portion of muscle mass, typically resulting from trauma, surgery, or degenerative diseases [1,2]. This condition severely impairs mobility, functionality, and quality of life while imposing a significant burden on healthcare systems worldwide. Unlike minor muscle injuries, which can heal through the activation of satellite cells and the subsequent repair of damaged fibres, VML exceeds the body's natural regenerative capacity, resulting in fibrosis and incomplete recovery [1,3]. Conventional therapeutic strategies, including autologous tissue grafting and prosthetic interventions, are often limited by factors such as donor site morbidity, restricted availability, and suboptimal restoration of functional outcomes [4,5].

Three-dimensional (3D) scaffold-based approaches have become well-established in regenerative medicine due to their ability to mimic the structural, mechanical, and biochemical properties of native tissues [[6], [7], [8]]. However, in the context of critical-size defects such as VML, these strategies alone are often insufficient to achieve complete tissue regeneration of excitable tissues such as skeletal muscle due to the complex bioelectrical and biomechanical demands involved.

Recent advancements have shifted attention toward integrating scaffolds with external stimuli, the so-called four-dimensional (4D) scaffold-based approaches, to further enhance regenerative outcomes [[9], [10], [11], [12], [13]]. These stimuli, which may include electrical, magnetic, or enzymatic cues, can deliver precise temporal and spatial signals to resident or transplanted cells, thereby modulating cellular behaviour and promoting tissue repair [[14], [15], [16], [17]]. Among the various external stimuli explored, electrical stimulation is one of the most extensively studied and promising approaches for musculoskeletal tissue regeneration. This technique, which applies controlled electrical currents to activate muscle fibres and promote cellular regeneration, is already used in clinical settings as a rehabilitation tool and may be applied transcutaneously or intramuscularly (e.g. neuromuscular electrical stimulation, NMES; transcutaneous electrical stimulation; TES) [[18], [19], [20]]. Bioelectrical signalling has gained considerable attention due to its critical role in orchestrating cell patterning, migration, and differentiation at both the cellular and tissue levels. Stimuli that intrinsically activate or modulate these signals can significantly enhance regenerative outcomes [21]. While it can enhance muscle activation and support functional recovery, electrical stimulation alone is insufficient to fully address the extensive tissue loss and functional impairments characteristic of VML [3].

To address this limitation, the development of electroconductive scaffolds has emerged as a promising strategy. These scaffolds, composed of conductive polymers (e.g. polypyrrole, polyaniline, PEDOT:PSS), carbon-based nanomaterials (e.g. graphene, carbon nanotubes), metal nanoparticles (e.g. gold), or their composites, can deliver localised electrical stimulation directly at the injury site, thereby enhancing cellular responses and promoting tissue regeneration [[22], [23], [24], [25]]. Nevertheless, many electrical stimulation systems rely on the direct coupling method, where conductive electrodes are in physical contact with the target tissue or conductive scaffold [26]. While this approach is widely used due to its straightforward implementation, it typically requires physical wiring that extends externally from biomaterials to an external power supply [27]. This configuration poses significant clinical challenges, particularly for deep or complex lesions, due to risks of infection, discomfort, and limited patient mobility. Alternative strategies, such as capacitive and inductive coupling, have been developed to overcome the limitations of direct contact by enabling wireless stimulation, but direct coupling remains the most prevalent method in clinical and research applications [28].

In this study, we explore a scaffold-based strategy for wireless electrical stimulation in skeletal muscle regeneration, leveraging the combination of egg white proteins (EWP) and barium titanate (BTO) piezoceramic particles. The mechanical deformation of embedded piezoelectric particles, induced by external ultrasound (US), generates localised electric cues to stimulate cellular responses. While US-driven activation of piezoelectric materials has been previously reported [16,29], the present work is distinguished by the integration of BTO within a biodegradable EWP matrix and by its application in a muscle regeneration context. In detail, we present a cost-effective and rapid microwave (MW)-assisted fabrication technique to develop egg white proteins (EWP)-based scaffolds integrated with piezoceramic particles of barium titanate (BTO). This method allows precise modulation of both the mechanical properties and the BTO content within the scaffold matrix. The resulting composite scaffolds harness the biocompatibility and tuneable characteristics of EWP alongside the piezoelectric capabilities of BTO. Upon exposure to external US stimulation, these scaffolds generate localised electrical signals, modulating Ca^2+^-dependent signalling pathways boosting the muscle cell proliferation and differentiation. This synergistic approach not only provides structural support but also facilitates dynamic bioelectrical modulation, offering a promising, non-invasive strategy to enhance muscle tissue repair, particularly in the context of VML. Through comprehensive *in vitro* evaluations, including detailed physicochemical and mechanical characterization, as well as assessment of piezoelectric and biological performances, this work aims to establish a multifunctional 4D scaffold system that addresses current limitations in regenerative therapies, paving the way for innovative, sustainable, and clinically viable solutions in musculoskeletal tissue regeneration.

## 2 Materials and methods

### 2.1 Piezoelectric scaffolds synthesis

The egg white proteins (1 kg bricks, Eurovo Srl, Italy) were allowed to reach laboratory temperature and were pre-foamed using a balloon whisk in a Nalgene beaker. Barium titanate powders (BaTiO_3_, 99% purity; average particle size ≈ 700 nm, Merck Sigma-Aldrich), exhibiting a tetragonal phase and submicrometric particle size (Fig. SI 1), were used as the starting piezoelectric active phase. BTO particles were gently incorporated into the foam to prevent lump formation, achieving the desired EWP:BTO ratios of 1:1, 1:2, and 1:4. The resulting composite mixtures were cast into silicone molds (5 mm diameter, 3 mm height) and subjected to microwave-assisted heat treatment for sintering, 10 min at 90 W followed by 1 min at 700 W, during which secondary foaming occurred due to microwave-induced expansion.

### 2.2 FTIR analysis

The Fourier transform infrared spectroscopy in attenuated total reflectance mode (ATR-FTIR) was performed using a PerkinElmer Spectrum 2 instrument acting with 16 scans in the range of 4000 to 400 cm^−1^ and a resolution of 0.5 cm^−1^. Before performing the FTIR analysis, the liquid EWP was heat treated in a MW oven to obtain the solid phase, following the same procedure as for the composite samples. A drop of EWP was placed on a plate and microwave-treated at 700 W for 1 min to obtain the solid phase. Other precursors were analysed in powder form, along with the three composites, which required MW heat treatment for sintering and removal of egg water.

### 2.3 Degradation test

To evaluate scaffold stability over time under physiological conditions, a degradation test was performed. The scaffolds were incubated in PBS 1X (Gibco) at 37°C and weighed at different time points up to 30 days: 0 d, 1 d, 3 d, 7 d, 10 d, 14 d, 18 d, 21 d, 24 d and 30 d. The degradation percentage (*D*) was calculated as follows:

(1) $$D\left(\%\right)=\left(\frac{W_{i}-W_{f}}{W_{i}}\right)x\;100$$

where *W_i_* is the initial weight right after the immersion in PBS 1X and *W_f_* is the weight at the selected time point. The data are reported as a percentage respect to the 0 d time point (*n* = 4).

To evaluate protein release associated with scaffold degradation, a protein quantification assay was performed on the supernatants collected at each degradation time point.

Protein content was quantified using a Lowry assay (DC protein assay kit, Bio-Rad), according to the manufacturer's instructions. Absorbance was measured at 750 nm using a microplate reader (Multiskan FC, Thermo Scientific), and protein concentrations were determined using a standard curve generated from known concentrations of bovine serum albumin (BSA, PAA).

### 2.4 Scaffold porosity evaluation

The porosity of the scaffolds was evaluated both qualitatively, using Scanning Electron Microscopy (SEM) (FEI Quanta 200, ESEM), and quantitatively, using X-ray micro-computed tomography (micro-CT).

For SEM analysis, the scaffolds were sputter-coated with gold using a Polaron Sputter Coater E5100 and examined at an accelerating voltage of 10 kV under high vacuum.

A more detailed assessment of the three-dimensional pore architecture was carried out by micro-CT. To enhance scaffolds imaging, phosphotungstic acid (PTA) (Sigma-Aldrich) was used as a contrasting agent. The staining was performed according to the adapted protocol of Kwon et al. [30]. Therefore, a solution of 1% (w/v) PTA-solution was prepared with distilled water. Each scaffold was immersed into 2 ml of PTA-staining solution and the reaction occurred at room temperature, overnight. After the staining, the scaffold was washed with distilled water and air-dried for further analysis. Digital radiographs were acquired with a micro-CT scanner (SkyScan 1172, Bruker, USA), by rotating the sample over 180° with a fixed 0.9° step, a source voltage of 50 kV and a source current of 800 μA. Experimental conditions were set to optimized acquisition time and best image contrast, with pixel size resolution of 9.55 μm and an average of three radiographs per position. Slice reconstruction of the raw scanned data was carried out with the NRecon®1.6.3 software. During the reconstruction, the value of ring artefacts reduction was set to 11%, smoothing to 2%, and the value of beam-hardening correction to 30%. 3D model visualization was obtained using the CTVox software (Bruker). 3D analysis (total porosity, pore size and distribution; *n* = 3) was performed with the CT-Analyser v1.20 software.

### 2.5 Dynamic mechanical analysis

Dynamic Mechanical Analysis (DMA) was conducted using a Q800 dynamic mechanical analyzer (TA Instruments) on EWP:BTO 1:1; EWP:BTO 1:2 and EWP:BTO 1:4 samples in order to determine their Young's modulus values. Samples were preconditioned by an overnight immersion in PBS 1X at 37°C. All the analyses were carried out at 37°C in PBS immersion. The Young modulus was measured in compressive mode, and a stress-strain test was performed to obtain the slope of the linear fit in the range of 0% to 10% (*n* = 5).

### 2.6 Corona poling and piezoelectric testing

The samples were electrically poled at room temperature in a custom build Corona discharge setup [31], using an electrode distance of 5 cm from the sample surface, 20 kV voltage and a poling duration of 5 min. Samples were poled in groups of six. The direct piezoelectric response was determined for five samples randomly chosen throughout the whole batch with a Berlincourtmeter (YE2730A *d*_33_-m, Sinocera Piezotronics Inc., China), applying 0.25 N at 100 Hz.

### 2.7 Transverse piezoresponse of scaffolds

Transverse piezoelectric coefficients *d*_31_ of the EWP:BTO 1:1 scaffolds were measured by a home-made apparatus named PiezoGauge [32]. This setup was specifically conceived to measure the piezoelectric yield of compliant samples in film geometry, with no need to deposit conductive electrodes on the film surface. This feature is especially useful for porous materials, where electrode deposition could easily lead to short circuits. The measurement sensitivity is better than 1 pm/V, allowing estimation of very low piezoelectric response. In brief, the instrument combines non-contact electrical polarisation of the sample by means of two parallel slabs located very close (but not touching) the film surfaces, and a resonant detection of the piezoelectric stress able to increase the signal-to-noise ratio of the measurement of around 5 times compared to a non-resonant measurement. A built-in calibration method allows to obtain quantitative evaluation of the transverse piezoelectric effect on samples with virtually any kind of mechanical properties, included very compliant ones like nanofibre meshes produced by electrospinning [33]. This calibration method compares electrically induced piezoelectric stress with a known mechanical stress generated by a calibrated piezoelectric transducer. This element is a piezoceramic slab, with piezoelectric coefficient *C*_P2_ (500 pm/V), connected in series with the sample. By virtue of this calibration method, the value of *d*_31_ is derived by the following formula:

(2) $$d_{31}=\frac{{\Delta A}_{E}}{\Delta A_{M}}\;\frac{C_{P2}V_{M}\eta \varepsilon_{s}\;}{V_{\text{ac}}l_{\eta }}$$

where Δ*A*_E_ is the oscillation amplitude detected when electrical excitation is applied, Δ*A*_M_ is the amplitude obtained with mechanical excitation, *V*_M_ is the ac voltage supplied to the piezoelectric transducer, *η* is the distance between the electrodes, *V*_ac_ is the *ac* voltage supplied to the electrodes, *l*_*η*_ is the length of the sample subjected to the electric field, and *ε*_s_ is a reduction factor for the electric field due to the employed geometry, that amounts to:

(3) $$\varepsilon_{s}=\varepsilon_{d}-\frac{\eta_{d}}{\eta }\left(\varepsilon_{d}-1\right)$$

where *ε*_d_ the dielectric constant of the sample, and *η*_d_ its thickness.

Dielectric constants of the EWP:BTO scaffolds, used to determine *ε*_s_, were measured on pressed pellets of the crushed materials by Broadband Dielectric Spectroscopy, as described in the Supporting Information.

Samples were produced in the form of elongated bars with around 40 mm length, around 5 mm width, and 1-2 mm thickness, in order to fit the PiezoGauge clamps. By weighing of the sample, an apparent density was derived, that led to a value of effective thickness (as if the sample was compact, with no cavities), used as the *η*_d_ value to determine *ε*_s_. The features of the measured samples are reported in Table 1.

**Table 1:** Geometrical features and derived useful quantities of the measured samples.

Sample	Length (mm)	Width (mm)	Thickness (mm)	Weight (mg)	Apparent density (kg/m^3^)	Effective thickness (mm)	Dielectric constant *ε*_s_ @222 Hz
EWP:BTO 1:1 Not poled	41.5	4	2	247.7	746	0.606	155
EWP:BTO 1:1 Poled	36	5	2	325.0	902	0.732	194

### 2.8 *In vitro* biological study

The C2C12 mouse myoblast cell line (ATCC® CRL-1772™) was maintained in complete growth medium (GM) composed of DMEM high glucose medium (Gibco) supplemented with 10% Fetal Bovine Serum (FBS, Gibco) and 1% Penicillin and Streptomycin solution (pen/strep, 100 U/ml-100 μg/ml, Gibco). For myogenic differentiation, cells were cultured in DMEM high glucose containing 2% horse serum and 1% pen/strep (differentiation medium, DM). All cell cultures were kept at 37°C under 5% CO_2_ atmosphere conditions and controlled humidity. Cells were harvested by trypsinization, and after centrifugation, the cell count and viability were assessed using the Trypan Blue Dye exclusion test.

For the biological evaluation, each scaffold (5 mm in diameter, 1.5 mm in height) was sterilized with 70% ethanol, followed by ultraviolet (UV) irradiation for 5 min in PBS 1X under a sterile laminar-flow hood. Subsequently, the samples were pre-conditioned by overnight incubation in a 48-well plate in complete cell culture medium at 37°C. The pre-conditioned scaffolds were seeded by carefully dropping 10 μl of cell suspension (1.0 × 10^4^ cells) on the top surface, followed by a 20-min incubation to promote cell attachment before the addition of 500 μl of complete growth medium. All cell-handling procedures were performed under biological laminar-flow hood and sterile conditions. The cell medium was replaced every three days until the end of the experiment.

#### 2.8.1 Cell viability and proliferation analysis

An initial biological screening was performed on the EWP:BTO 1:1; EWP:BTO 1:2 and EWP:BTO 1:4 groups. Quantitative evaluation of cell viability and proliferation over time was carried out by Thiazolyl Blue Tetrazolium Bromide (MTT) assay at day 1, 3 and 7 of culture according to manufacturer's instructions. Briefly, at each time point, each sample was incubated with 10% (v/v) MTT solution (Merck, 5 mg/ml) in culture medium for 2 h at 37°C, 5% CO_2_ atmosphere and controlled humidity conditions. The metabolically active cells reacted with the tetrazolium salt in the MTT reagent to produce formazan crystals. Then, scaffolds were transferred to a tube containing 1000 μl of dimethyl sulfoxide (DMSO, Sigma Aldrich) to dissolve the insoluble formazan crystals derived from MTT conversion. After 15 min of incubation, the supernatants were collected and analysed using a UV-visible spectrophotometer (Multiskan FC, Thermo Scientific) by measuring absorbance at 570 nm. Four samples for each group were evaluated (*n* = 4), and the corresponding blank average (scaffold with no cells seeded) was subtracted from each measurement. MTT test was also performed afterwards on EWP:BTO 1:1 poled and not poled samples, at day 3, 5 and 7 of culture, following the procedure described above.

A qualitative analysis to evaluate C2C12 cell viability seeded on the EWP:BTO 1:1; EWP:BTO 1:2 and EWP:BTO 1:4 scaffolds was performed on day 7 using LIVE/DEAD® assay following the manufacturer's instructions. Briefly, cells were washed with PBS 1X and incubated with 1.3 μM of Calcein AM and 4 μM of Ethidium homodimer-1 for 15 min at 37°C and 5% CO_2_. Images of live cells stained in green and dead cells in red were acquired by using an Inverted Ti-E fluorescent microscope (Nikon). One sample for each group was analysed (*n* = 1).

#### 2.8.2 Cell morphology evaluation

The morphology of C2C12 cells on EWP:BTO 1:1, both poled and not poled; EWP:BTO 1:2, and EWP:BTO 1:4 scaffolds was visualized by fluorescent staining of actin filaments and by scanning electron microscopy (SEM).

For actin staining, at day 7 of culture, samples were fixed with 4% (w/v) paraformaldehyde (PFA, Merck KGaA) for 15 min, followed by cell membrane permeabilized with 0.1% (v/v) Triton X-100 (Sigma Aldrich) in PBS 1X for 15 min at room temperature. Actin filaments were stained using Actin Red 555 ReadyProbes™ Reagent (Invitrogen) for 30 min, and cell nuclei were counterstained with DAPI (600 nM, Invitrogen) in PBS 1X for 7 min. Fluorescent images were captured using a Nikon Inverted Ti-E fluorescence microscope. One sample per group was analysed (*n* = 1).

A further qualitative cell morphology analysis was performed by SEM at day 7 of culture. Briefly, the samples were washed in 0.1 M Sodium Cacodylate Buffer (pH 7.4, Sigma Aldrich) and fixed in 2.5% Glutaraldehyde (Merck KGaA) solution in 0.1 M Sodium Cacodylate Buffer for 2 h at 4°C. Then, they were washed once with cacodylate buffer 0.1 M (pH 7.4) for 5 min and twice with MilliQ water for 10 min. After that, the samples were frozen at −20°C, freeze-dried, sputter-coated with gold, as described in Section 2.4, and analysed by SEM (FEI Quanta200, ESEM).

#### 2.8.3 Scaffold colonization

To evaluate cell penetration within the EWP:BTO 1:1 scaffold, both poled and not poled samples were fixed with 4% PFA at day 7 of culture in complete and differentiation media, stained with DAPI to visualize cell nuclei, and imaged using a Nikon Inverted Ti-E fluorescence microscope. For each experimental group, three scaffolds were analysed (*n* = 3). The samples were cut in half to expose the internal structure, and image acquisition was performed along the cross-sectional plane of the scaffold. For each scaffold half, multiple images were captured across the cross-section. In each image, 15 measurements of the cell penetration depth were taken using FIJI [34], an open-source image analysis software based on ImageJ, defined as the distance from the scaffold surface to the deepest visible DAPI-positive nucleus. The values were then expressed as a percentage of the total scaffold thickness to quantify relative cell colonization depth.

#### 2.8.4 Immunofluorescence analysis

An evaluation of cell adhesion was performed by immunofluorescent detection of Focal Adhesion Kinase (FAK) and Paxillin on EWP:BTO 1:1 poled and not poled scaffolds at day 7 of culture in complete and differentiation media.

Briefly, the samples were fixed in 4% (w/v) PFA for 15 min, then washed 3 times in PBS 1X for 5 min, followed by blocking in 1% BSA + 10% Normal Goat Serum (NGS, Euroclone, Italy) in PBS 1X for 30 min at room temperature under slow agitation. The cell membranes were permeabilized in PBS 1X with 0.1% (v/v) Triton X-100 for 15 min at room temperature. Then, after three more washes in PBS 1X, the primary antibodies against FAK (1:100, rabbit NBP2-67327, Novusbio) and against Paxillin (1:100, rabbit NBP2-67427, Novusbio) in 1% NGS in PBS 1X were added to the samples, and an overnight incubation at 4°C in humidity chamber was carried out.

A Goat anti-rabbit IgG antibody (1:500, Alexa Fluor® 488 goat anti-rabbit IgG H&L, Abcam) was used as secondary antibody in 1% NGS in PBS 1X for 1 h incubation at room temperature in the dark. DAPI staining (600 nM in PBS 1X, 10 min at room temperature) was used for cell nuclei detection. After three final washes in PBS 1X, the scaffolds were observed in a Nikon Inverted Ti-E fluorescent microscope. One sample for each group was analysed (*n* = 1).

#### 2.8.5 Ultrasound-mediated piezoelectric stimulation

**Fig. 1:**
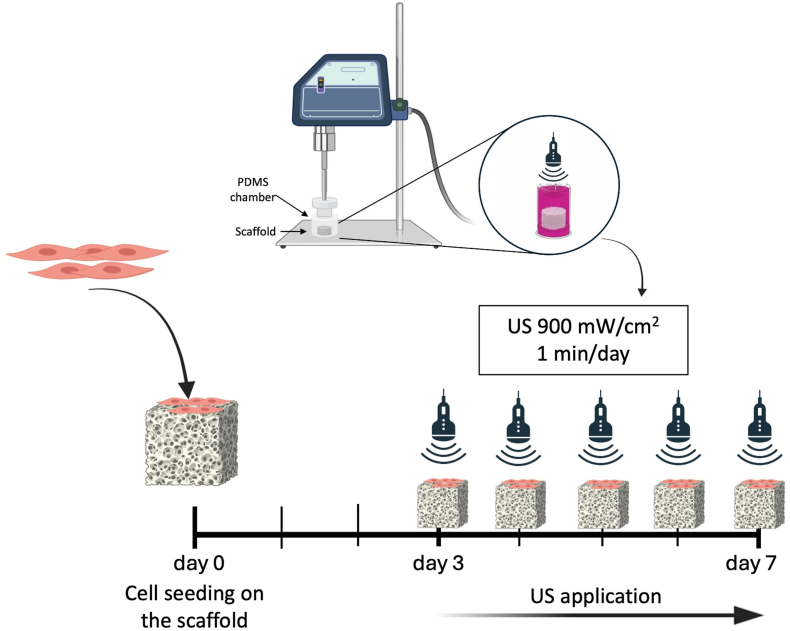
Experimental design of the ultrasound-mediated piezoelectric stimulation.

3 days after cells seeding on the EWP:BTO 1:1 poled and EWP:BTO 1:1 not poled scaffolds, ultrasound (US) stimulations were performed using a Vibra-Cell VCX 130 ultrasonic processor (Sonics & Materials Inc.) equipped with a 6 mm diameter probe, operating at 20% amplitude and a frequency of 20 kHz. To optimize US stimulation, a custom culture chamber was designed and manufactured by a mold-casting process of polydimethylsiloxane (PDMS, Sylgard® 184, Merck) as described in the Supporting Information. PDMS was selected for its favourable mechanical and acoustic properties, including an acoustic impedance comparable to that of cell culture media, making it suitable for effective ultrasonic transmission [35]. The experimental setup allowed for consistent and reproducible ultrasound exposure. An acoustic intensity of 900 mW/cm^2^ was applied [36]. EWP:BTO 1:1 scaffolds, both poled and not poled, were placed in the chamber, immersed in culture medium, and subjected to a 60-s ultrasound stimulation once daily for five consecutive days (Fig. 1; Fig. SI 7).

#### 2.8.6 Western blot analysis

After 7 days of culture on both poled and not poled scaffolds in either GM or DM, cells were lysed using radioimmunoprecipitation (RIPA) buffer supplemented with a protease inhibitor cocktail (Cell Signaling). The protein concentration in each cell lysate supernatant was quantified with a colorimetric assay (DC Protein Assay kit, Bio-Rad). Protein samples (*n* = 2 per group) were mixed with sample buffer at a 3:1 ratio, loaded in 4–20% Mini-PROTEAN TGX stain-free protein gels (Bio-Rad), and separated by electrophoresis using a Mini-PROTEAN electrophoresis cell kit (Bio-Rad).

Proteins were then transferred to nitrocellulose membranes by means of a Trans-Blot Turbo™ transfer system (Bio-Rad). The membranes were blocked for 40 min at room temperature in 5% nonfat dry milk diluted in PBS 1X containing 0.1% Tween-20 (Bio-Rad), then incubated overnight at 4°C under soft agitation with the following primary antibodies: anti-FAK (1:1000, rabbit NBP2-67327, Novusbio), anti-Paxillin (1:1000, rabbit NBP2-67427, Novusbio), anti-Myogenin (1:250, mouse MA511486, Thermo Scientific) and anti-β-actin (1:400, mouse MA511869, Invitrogen) used as loading control. After 3 washes in PBS 1X + 0.1% Tween-20, the membranes were incubated with a horseradish goat peroxidase-linked secondary antibody (anti-rabbit or anti-mouse, Bio-Rad), for 1 h 30 min. An enhanced chemiluminescence kit (ECL, Bio-Rad) was used to visualize the protein bands with ChemiDoc XRS+ (Bio-Rad). To evaluate the relative protein expression, FAK, Paxillin and Myogenin band intensities were quantified by densitometry using ImageLab software and were then normalized over the signal of the corresponding β-actin bands.

#### 2.8.7 Gene expression analysis

After culturing C2C12 cells on the EWP:BTO 1:1 poled and EWP:BTO 1:1 not poled scaffolds for 7 days in both complete growth and differentiation media, the cells' gene expression profile was evaluated, using a 7-day 3D culture of C2C12 in complete medium as a control. Scaffolds were individually collected and mechanically dissociated using homogenizing pestles and Tri Reagent (Invitrogen). Total RNA extraction and purification were performed by using Tri Reagent and a purification kit (Direct-zol RNA MiniPrep kit, Zymo Research), following the manufacturer's instructions. RNA concentration and purity degree were determined with the NanoDrop One Microvolume UV–Vis Spectrophotometer (Thermo Scientific), according to the manufacturer's instructions. Complementary DNA (cDNA) was synthesized from 500 ng of purified RNA using the High-Capacity cDNA Reverse Transcription Kit (Applied Biosystems), following the manufacturer's guidelines. The resulting cDNA was analysed via Real-Time PCR using the TaqMan Gene Expression Assay Kit (Applied Biosystems). ITGβ3 (Mm00443980_m1), NFATc1 (Mm01265942_m1), Myogenin (Mm00446194_m1) and MyoD1 (Mm00440387_m1) genes were evaluated, and GAPDH (Mm99999915_g1) was used as housekeeping gene. Two independent experiments were performed with two samples per group each, using three technical replicates. Data were collected from the QuantStudio 1 Real-Time PCR System (Applied Biosystems) and the relative quantification of target gene was assessed with the Comparative Threshold (Ct) method (ΔΔCt), where relative gene expression level equals to 2^−ΔΔCt^ [37]. The not poled GM samples were used as the calibrator for relative gene expression analysis.

### 2.9 *In vivo* biological evaluation

C57/Bl6 mice (male; 14 weeks-old, *n* = 3) were used for EWP:BTO 1:1 not poled scaffolds subcutaneous implantation. Before surgery, buprenorphine (0.1 ml/10 g body weight) was administered to the animals. The animals were anesthetized by isoflurane inhalation, the dorsum was shaved and the surgical area was prepared with an aseptic technique. A midline incision at the dorsal skin was performed and 3 scaffolds per mice were subcutaneously implanted. After surgery, the mice received paracetamol dissolved in water. After 3, 7 and 28 days, mice were sacrificed with carbon dioxide inhalation and the scaffolds and surrounding tissues were processed for histological analyses. Potential systemic toxicity was evaluated in the kidney, liver and spleen. Animal experimentation was carried out at i3S - Instituto de Investigação e Inovação em Saúde animal facility, approved by the local Animal Ethics Committee and by Direcção Geral de Alimentação e Veterinária through licence n° 122395/24-S 22-10-2024 and conducted in accordance with the European Legislation on Animal Experimentation through Directive 2010/63/UE.

#### 2.9.1 Histological analysis

Explanted scaffolds and organs (kidney, liver and spleen) were collected, fixed in formalin 4% and processed for paraffin embedding. Serial sections of 5 μm thickness were obtained, deparaffinized, and rehydrated through a series of washes in xylene, 100% ethanol, 80% ethanol, and MilliQ water. Sections were then stained using Haematoxylin and Eosin. Briefly, the sections were incubated with Mayer Haematoxylin (Sigma Aldrich) with added acetic acid for 3 min at room temperature and washed for 10 min in running water followed by a wash in MilliQ water. Eosin Y (Sigma Aldrich) was then added to the sections for 3 min and washed in MilliQ water.

### 2.10 Statistical analysis

All the results were plotted as mean ± standard error of the mean (SEM), and statistical analyses were performed by GraphPad Prism Software (Version 8.0). All C2C12 proliferation data were analysed by Two-way analysis of variance (Two-way ANOVA), followed by Sidak's and Tukey's Multiple Comparisons test. Young's moduli, gene expression and Western blot quantification data were analysed by One-way analysis of variance (One-way ANOVA), followed by Tukey's Multiple Comparisons test. Cell colonization data were analysed by unpaired *t*-test. Statistically significant differences are reported in the graphs: ∗p value ≤ 0.05, ∗∗p value ≤ 0.01, ∗∗∗p value ≤ 0.001 and ∗∗∗∗p value ≤ 0.0001.

## 3 Results and discussion

EWP have emerged as an unexpectedly versatile material platform, attracting attention not only in food science but increasingly in materials chemistry and biomedical engineering [38]. Their ability to act as foaming, gelling, emulsifying, and templating agents has been exploited to fabricate a wide variety of functional materials, while their sustainability, low cost, and intrinsic biocompatibility make them particularly appealing for regenerative applications [[38], [39], [40]]. Inspired by these unique attributes, we explored the use of EWP as a structural matrix for the fabrication of piezoelectric scaffolds. To introduce the piezoelectric functionality, BTO particles were incorporated as the active phase. This choice was motivated by its combination of structural versatility and exceptional physical properties. BTO is, in fact, a ferroelectric ceramic material with a perovskite structure well-known for its high dielectric constant and piezoelectricity. Because of these properties, it is widely used in capacitors, sensors, actuators, and electro-optic devices [41,42]. Importantly, its biocompatibility and favourable interactions with biological systems extend its potential to biomedical contexts [[43], [44], [45]]. Recent studies demonstrate that BTO can generate localised electrical cues that influence cell adhesion, proliferation, and differentiation, making it valuable for tissue engineering and bio-stimulation. Together, these features establish BTO as a multifunctional additive within EWP-based scaffolds, providing both structural support and active bioelectric modulation.

### 3.1 Fabrication of piezoceramic-integrated scaffolds

The fabrication process of scaffolds was optimized to exploit the natural foaming ability of EWP while ensuring the structural integrity of the composite. Preliminary tests identified the optimal microwave conditions required to balance expansion and thermal stability (data not shown). Excessive power led to localised burning of the protein phase, whereas insufficient power caused incomplete foaming and residual moisture. A two-step microwave treatment (i.e. 10 min at 90 W followed by 1 min at 700 W) was therefore selected as the most effective for achieving homogeneous expansion, complete dehydration, and intrinsic sterilization of the samples.

For the choice of BTO concentrations, the lowest selected ratio ensured that the composite exhibited a piezoelectric effect strong enough to be measurably detected, while the highest represented the maximum concentration at which the raw mixture remained minimally workable; at these higher loadings, the paste exhibited a very compact, toothpaste-like consistency, but could still be manipulated as required for moulding and processing.

Because egg white is approximately 90% water and 10% proteins, microwave processing resulted in the solidification of the protein network, which encapsulated BTO particles within a porous “meringue-like” structure with a homogeneous distribution of BTO particles.

During sample preparation, the pre-foaming (by balloon whisk) of EWP led to a tenfold increase in volume. When the lowest amount of BTO (1:1) was added, only a slight volume reduction was observed. In contrast, the addition of the largest amount of BTO (1:4) resulted in a significant volume decrease. This behaviour appears to correlate with the presence of inorganic powders, likely due to water absorption from the foamed egg white phase.

A second foaming process occurs during MW-oven heating. As a result, the 1:1 ratio, due to its higher water content, was expected to produce larger pores and greater overall porosity. Conversely, the 1:4 ratio was anticipated to yield fewer and smaller pores. This trend is supported by the SEM characterization, but it is not confirmed by the porosity measurements. A possible explanation is provided in the corresponding section.

### 3.2 Chemical, morphological, and mechanical evaluation of EWP:BTO scaffolds

**Fig. 2:**
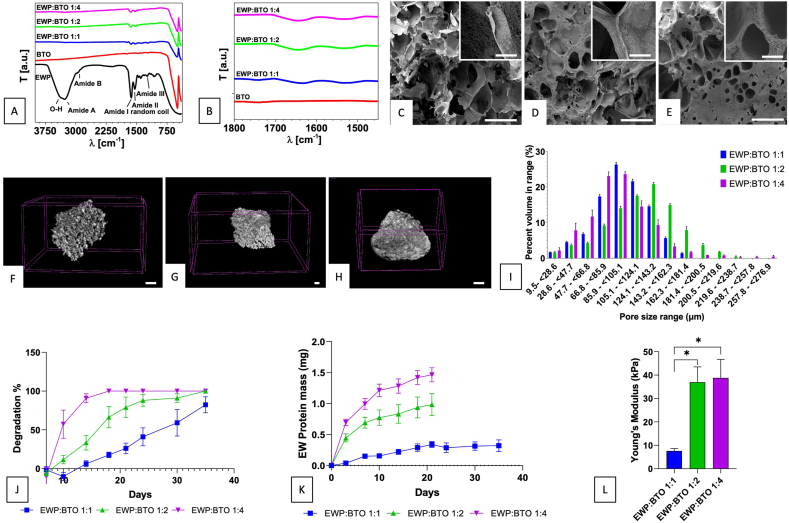
**A-B**. FTIR patterns, from the bottom to the top, of: MW heat treated EW proteins (black), BTO powder (red), EWP:BTO 1:1 composite (blue), EWP:BTO 1:2 composite (green) and EWP:BTO 1:4 composite (purple). **C-E**. Representative SEM images of EWP:BTO scaffolds prepared at different weight ratios: (C) 1:1, (D) 1:2, and (E) 1:4. Insets show higher magnification views highlighting the homogeneous distribution of BTO particles within the porous matrix. Scale bars: 500 μm, insets scale bars: 50 μm. **F-H**. Three-dimensional computed tomography model of the EWP:BTO 1:1 (F), EWP:BTO 1:2 (G), and EWP:BTO 1:4 (H) scaffolds. Scale bars: 500 μm. **I**. Pore size distribution of the scaffolds (mean ± SD, *n* = 3). **J**. Scaffold degradation profile over time, expressed as % weight loss relative to day 0 (*n* = 4). **K**. Protein release from the scaffold during degradation over time (in mg), quantified using a Lowry assay. Data are shown only while samples remained recoverable; no data for EWP:BTO 1:2 and 1:4 beyond 21 days due to the degradation (see graph J). **L**. Dynamic mechanical analysis scaffold characterization (Young's Modulus, *n* = 5); ∗p value ≤ 0.05.

As shown in Fig. 2A and B, the typical FTIR patterns of the composite EWP (black) and BTO powder (red) are reported together with the spectra of the binary EWP:BTO composites (blue, green, and purple lines) with compositions of 1:1, 1:2, and 1:4, respectively. The black line represents the spectrum of pure egg white. The broad peak observed between 2850 and 3750 cm^−1^ is formed by two peaks with maxima at 3360 and 3260 cm^−1^, corresponding to residual H_2_O and the N–H stretching vibration of the amide A group, respectively. Within this range, a shoulder at 2978 cm^−1^ can also be identified, associated with the CH_2_ asymmetric stretching of the amide B group. The intense peak lying at 1640-1648 cm^−1^ corresponds to the stretching of C=O group, and its position indicates the random coil configuration of the amide group (amide I). The presence of amide II (in plane bending of N-H and stretching of C-N) and III (stretching of N-H) is indicated by the peaks at 1500-1600 cm^−1^ and 1200-1300 cm^−1^ [46,47]. The red line represents the FTIR pattern of the piezoelectric ceramic BTO, which shows its typical identification peaks corresponding to the stretching of Ti-O bond in the octahedron [Ti-O_6_]. No significant impurities are detected, although very small peaks between 1730 and 1210 cm^−1^, possibly due to carbonates used in BTO synthesis, can be observed [48,49]. For all three composites, the presence of BTO is confirmed by the characteristic absorption bands at low wavenumbers (<750 cm^−1^), while the typical fingerprints of the amide groups are clearly visible between 1500 and 1600 cm^−1^.

An initial screening of scaffold morphology was performed on the samples using SEM. The analysis confirmed that the intrinsic foaming properties of EWP promoted the formation of a porous network in which BTO particles were evenly embedded within the protein matrix (Fig. 2C–E). The morphology of the scaffolds was consistent across the different formulations, although increasing the BTO content from 1:1 to 1:4 (EWP:BTO) resulted in a denser distribution of inorganic particles within the pores, as clearly visible in the representative images (Fig. 2C–E insets). Notably, the meringue-like architecture generated by the microwave-assisted foaming process was preserved at all compositions, supporting the robustness of the fabrication strategy.

To complement the SEM observations, micro-CT was performed to provide a three-dimensional quantitative assessment of pore morphology and particle distribution. This analysis allowed us to evaluate pore size, interconnectivity, and the spatial homogeneity of BTO dispersion within the scaffolds, thereby offering a comprehensive structural characterization beyond surface-level imaging. Micro-CT analysis revealed that all scaffold formulations exhibited a heterogeneous and interconnected porous network, although clear differences emerged among the three compositions. Quantitative porosity measurements indicated that EWP:BTO 1:2 displayed the highest total porosity (47.38 ± 5.87%), followed by EWP:BTO 1:4 (37.64 ± 0.23%), whereas EWP: BTO 1:1 showed the lowest value (35.36 ± 1.96%) (Table 2). The pore size distribution analysis further highlighted such differences: EWP:BTO 1:2 exhibited a broad distribution, with the majority of pores ranging between 105.1 and 143.2 μm; EWP:BTO 1:1 displayed an intermediate profile, with most pores between 85.9 and 105.1 μm; and EWP:BTO 1:4 presented a narrower distribution, dominated by smaller pores in the range of 66.8–105.1 μm (Fig. 2F–I; Video SI 1-3). These architectural variations can be attributed to the influence of BTO content on the foaming dynamics of the EWP matrix. At the intermediate ratio EWP:BTO 1:2, BTO particles promote bubble nucleation and foam stabilization, favouring the formation of a highly porous and heterogeneous structure. In contrast, the higher particle content in EWP:BTO 1:4 scaffold likely increases the viscosity of the system and induces particle agglomeration, limiting foam expansion and leading to denser, less uniform porosity. EWP:BTO 1:1 group showed an intermediate behaviour, consistent with a reduced influence of the inorganic phase on the foaming process.

**Table 2:** Mean porosity (%) ± Standard Deviation (SD) of the samples (*n* = 3), as determined by micro-CT analysis.

	Total Porosity (%)
EWP:BTO 1:1	35.36 ± 1.96
EWP:BTO 1:2	47.38 ± 5.87
EWP:BTO 1:4	37.64 ± 0.23

When incubated in PBS 1X at 37°C, all scaffolds maintained their structural integrity for up to 10 days. Among them, the EWP:BTO 1:1 group exhibited the most gradual degradation profile, remaining stable for 21 days, with complete degradation observed by day 35 (Fig. 2J; Fig. SI 8). Quantitative analysis of protein release from the supernatants collected at each degradation time point revealed a clear correspondence between degradation kinetics and protein release (Fig. 2K). The EWP:BTO 1:1 scaffold showed the lowest cumulative protein release over time, consistent with its slower degradation rate. In contrast, scaffolds with higher BTO content (1:2 and 1:4) showed significantly higher levels of protein release at earlier time points, consistent with their faster degradation kinetics.

This behaviour may be partially related to differences in scaffold architecture observed by micro-CT. The 1:1 composition, showing an intermediate porosity and more uniform pore size distribution, likely provides a more cohesive structure that could better resist rapid fluid infiltration and dissolution. The controlled degradation and reduced protein release of the 1:1 scaffold indicate improved structural stability, making this composition the most suitable for applications requiring prolonged integrity during the early phases of cell colonization and tissue remodelling [[50], [51], [52]].

Mimicking the mechanical properties of the target tissue is particularly important from a biomimetic perspective, as it helps recreate a supportive cellular niche that can promote effective and functional tissue regeneration. In this study, the EWP:BTO 1:1 scaffold exhibited the lowest Young's modulus (7.6 ± 1.9 kPa), a value comparable to that of native musculoskeletal tissues [[53], [54], [55], [56]], whereas the EWP:BTO 1:2 and EWP:BTO 1:4 scaffolds displayed considerably higher stiffness, with Young's moduli of 36.9 ± 14.6 kPa and 38.8 ± 15.8 kPa, respectively (Fig. 2L). The moderate stiffness of the 1:1 scaffold, combined with its favourable structural characteristics (e.g. intermediate porosity, relatively uniform pore distribution, and controlled degradation profile), supports its mechanical and biological suitability for soft tissue applications.

### 3.3 *In vitro* biological screening of EWP:BTO scaffolds

**Fig. 3:**
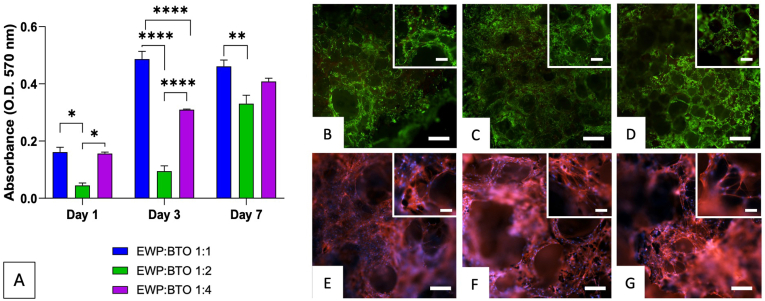
**A**. Cell proliferation measured by MTT test after 1, 3 and 7 days of culture (mean ± SEM, *n* = 4). ∗p value ≤ 0.05, ∗∗p value ≤ 0.01, and ∗∗∗∗p value ≤ 0.0001, where not indicated, differences are not statistically significant. **B-D**. LIVE/DEAD assay to assess cell viability at after 7 days of culture on EWP:BTO 1:1 (B), EWP:BTO 1:2 (C) and EWP:BTO 1:4 (D) samples. Live cells in green; dead cells in red. **E-G.** Morphological evaluation on C2C12 cells after 7 days of culture on EWP:BTO 1:1 (E), EWP:BTO 1:2 (F) and EWP:BTO 1:4 (G) samples. Actin filaments in red; cell nuclei in blue. Scale bars: B-D 500 μm, insets scale bars: 200 μm; E-G 200 μm, insets scale bars: 50 μm.

An initial biological screening was performed on the EWP:BTO 1:1, 1:2, and 1:4 scaffolds. *In vitro* cellular response was assessed using C2C12 murine myoblasts seeded on top of the scaffolds and cultured for up to 7 days. Quantitative evaluation of cell viability and proliferation was carried out by MTT assay. All scaffold compositions supported C2C12 viability and proliferation throughout the culture period, with a gradual increase in metabolic activity observed from day 1 to day 7 (Fig. 3A). Among the tested formulations, EWP:BTO 1:1 exhibited the most pronounced early growth, with metabolic activity increasing rapidly from day 1 to day 3 and remaining stable until day 7. This suggests that the EWP:BTO 1:1 composition provides a more favourable microenvironment for initial cell attachment and proliferation compared to the 1:2 and 1:4 scaffolds. These results are consistent with the scaffold's moderate porosity, uniform pore distribution, controlled degradation, and mechanical properties, which together likely facilitate nutrient diffusion and cell colonization while maintaining structural support. A further qualitative analysis using LIVE/DEAD® assay at day 7 corroborated these findings. Most cells colonising all three scaffolds were viable and well distributed throughout the scaffold pores (Fig. 3B–D).

An analysis of cell morphology was performed using fluorescent staining of the actin cytoskeleton. The results showed that C2C12 cells successfully adhered to and colonized the surface of all three scaffold compositions, with clear appreciation of the three-dimensional architecture (Fig. 3E–G). Actin staining revealed well-spread, elongated cells characteristic of myoblasts, with the formation of an interconnected cellular network throughout the scaffolds. Although all three scaffold compositions supported C2C12 viability and exhibited favourable cell morphology, based on the quantitative proliferation data and the previously described favourable porosity, controlled degradation, and compliant mechanical properties, the EWP:BTO 1:1 scaffold was selected for further in-depth *in vitro* and subsequent *in vivo* studies.

### 3.4 Scaffold polarisation analysis

To enhance the piezoelectric functionality of the scaffold, the EWP:BTO 1:1 samples were subjected to corona poling, a high-voltage, non-contact treatment used to align the dipoles of the embedded BTO particles. This process imparts a preferential orientation to the piezoelectric phase, enabling the scaffold to generate higher localised electric signals under mechanical stimulation. The direct piezoelectric *d*_33_ coefficient determined after poling was −2.6 ± 0.4 pC/N, while not poled samples presented an overall negligible net charge.

Transverse piezoelectric *d*_31_ measurements were conducted on six different EWP-BTO 1:1 scaffolds, three before and three after poling. The results on not poled samples were 0.037 ± 0.003, 0.035 ± 0.007, and 0.9 ± 0.1 pm/V, with an average of 0.32 pm/V, while those on the poled ones were 0.13 ± 0.01, 0.008 ± 0.003, and 0.38 ± 0.04 pm/V, with an average of 0.17 pm/V. The resulting average on the six samples resulted 0.25 pm/V. Within the large sample-to-sample variability, no clear increase in *d*_31_ after poling can be identified. This suggests that, in these scaffolds, the transverse piezoelectric response may be affected by the microstructure of each sample. This behaviour differs from that observed for *d*_33_, which was more clearly affected by corona poling. A possible explanation is that corona poling mainly promotes a net polarisation component along the direction of the applied electric field, whereas the transverse response remains more sensitive to the random orientation of the orthogonal components of the ferroelectric domains, that are not preferentially oriented by corona poling and therefore may exhibit partial cancellation. As a result, the average *d*_31_ value was approximately 10% of the *d*_33_ measured on poled samples. However, because of the limited number of samples and the strong influence of one high unpoled value, these results should be interpreted as preliminary.

### 3.5 *In vitro* biological assessment of poled EWP:BTO 1:1 scaffold

To evaluate the influence of polarisation on the bioactivity of the EWP:BTO 1:1 scaffold, a more in-depth *in vitro* biological study was conducted by comparing poled and not poled scaffolds. Specifically, polarisation aligns the internal dipoles of the BTO particles, generating two stable surfaces of opposite polarity. In literature, there are reports indicating that material polarisation induces permanent surface charges, which can positively influence cellular behaviour. However, it remains controversial which surface polarity is more effective, as different effects have been reported [[57], [58], [59]]. Nevertheless, some promising results have been observed with the positive surface [60,61]. For this study, we chose to begin our investigation by using the positive surface as the seeding side for C2C12 cells, based on these findings.

**Fig. 4:**
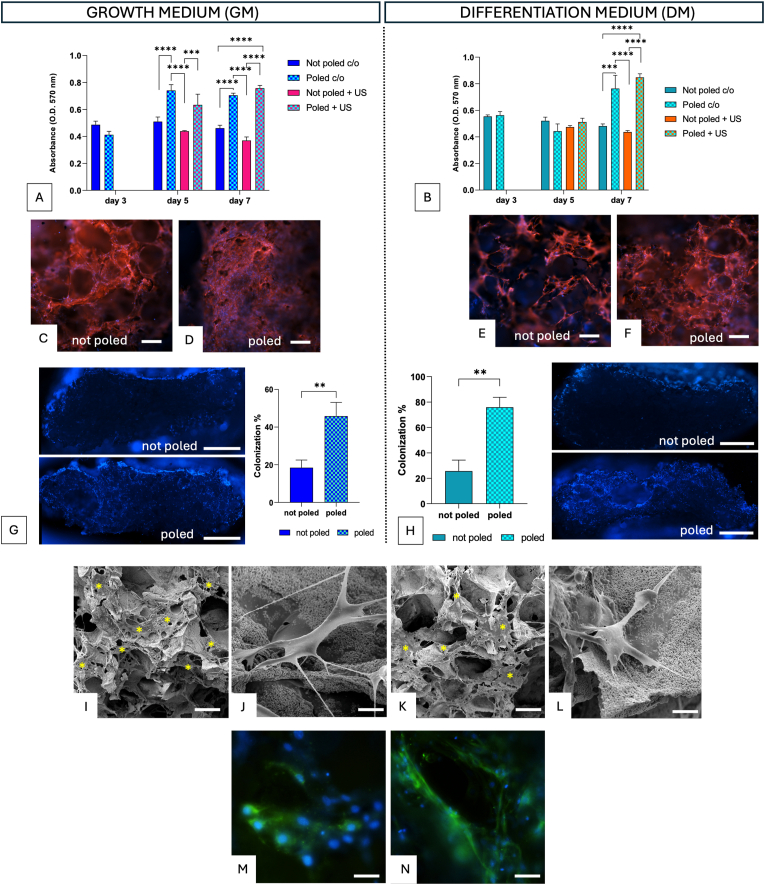
**A-B**. Cell proliferation measured by MTT test after 3, 5 and 7 days of culture (mean ± SEM, *n* = 4). US stimulation was initiated after 3 days of culture and applied daily until day 7, as outlined in Fig. 1; therefore, US-treated groups are not shown at the day 3 time point. **C-F**. Morphological evaluation on C2C12 cells after 7 days of culture on poled and not poled EWP:BTO 1:1 scaffolds in GM or DM. Actin filaments in red; cell nuclei in blue. **G-H**. Cell colonization of poled and not poled EWP:BTO 1:1 scaffolds in GM or DM (*n* = 3). Graphs: quantitative analysis of cell penetration depth, expressed as % of total scaffold thickness. Images: representative merged fluorescence showing cell distribution across the scaffold cross-section (cell nuclei in blue). **I-L**. SEM images of cell morphology on EWP:BTO 1:1 scaffolds in GM (I-J) and DM (K-L) after 7 days of culture. Yellow asterisks highlight the cellular network. **M**. Immunofluorescence detection of FAK protein in cells cultured for 7 days on not poled EWP:BTO 1:1 scaffolds in DM. FAK in green, cell nuclei in blue. **N**. Immunofluorescence detection of Paxillin protein in cells cultured for 7 days on poled EWP:BTO 1:1 scaffolds in GM. Paxillin in green, cell nuclei in blue. ∗∗p value ≤ 0.01, ∗∗∗p value ≤ 0.001 and ∗∗∗∗p value ≤ 0.0001, where not indicated, differences are not statistically significant. Scale bars: C-F 200 μm; G-H 1 mm; I-K 150 μm; J-L 25 μm; M − N 50 μm.

Cells were cultured onto the scaffolds up to 7 days, both in complete growth medium (GM) and differentiation medium (DM) conditions. A particularly relevant finding from the cell proliferation analysis is the significant difference in cell growth between poled and not poled scaffolds (Fig. 4A and B). In both conditions, complete growth medium and differentiation medium, cells proliferated more on the poled scaffolds. Specifically, in GM, a significant increase in cell viability was observed on poled scaffolds starting from day 5 of culture, compared to not poled ones (p value ≤ 0.001). Similarly, in DM, cell viability on day 7 was significantly higher on poled scaffolds compared to not poled ones (p value ≤ 0.001).

A qualitative morphological investigation was performed by SEM and actin cytoskeleton staining on C2C12 cells cultured on the EWP:BTO 1:1 scaffold (poled and not poled) after 7 days of culture. Immunofluorescent staining of actin filaments confirmed that the C2C12 cells adhered to and colonised the 3D scaffold architecture (Fig. 4C–F). Actin cytoskeleton labelling revealed elongated, well-spreading myoblasts forming a connected cellular network throughout the scaffold pores. No significant qualitative morphological difference in cell shape or cytoskeletal organisation was detected between the poled and not poled groups, reinforcing the conclusion that both scaffold conditions support favourable cell colonization. The only difference lies mainly in cell density rather than cell morphology, in agreement with the quantitative cell viability data from the MTT assay. SEM analysis revealed that the myoblasts were well adhered and extensively spread on the scaffold surfaces, establishing intimate contact with the underlying matrix without noticeable differences between the groups (Fig. 4I and J). In particular, one representative image shows a dense network of interconnected cells spanning across the pores of the scaffold and forming a continuous cellular layer (Fig. 4I), while a higher magnification detail reveals a single myoblast in tight adhesion to the scaffold, demonstrating direct cell–matrix interface contact (Fig. 4J).

To assess whether scaffold polarisation could influence cell adhesion and migration, a cell colonization assay was performed. Cell penetration within the EWP:BTO 1:1 scaffolds, both poled and not poled, was evaluated after 7 days. Cross-sectional imaging of DAPI-stained scaffolds revealed that cells penetrated deeper into the poled EWP:BTO 1:1 scaffolds compared to the not poled ones, under both growth and differentiative culture conditions (Fig. 4G and H). Quantitative analysis confirmed that the mean penetration depth, expressed as a % of the total scaffold thickness, was significantly higher in the poled groups. Specifically, after 7 days of culture in GM, cells infiltrated 45.8 ± 7.2% of the scaffold thickness in the poled samples, whereas only 18.5 ± 4.1% penetration was observed in the not poled controls (p value ≤ 0.01). A similar outcome was observed in the DM samples, where the poled one showed a 75.9 ± 7.8% cell penetration, while the not poled one showed only 25.7 ± 8.6% (p value ≤ 0.01).

These results demonstrate that, despite both scaffold types possess comparable porosity suitable for cell colonization, polarisation exerts a positive influence on cell adhesion and migration, likely through enhanced electrostatic interactions between the positively charged scaffold surface and the negatively charged cell membrane [60,61]. Improved cell infiltration is essential for the formation of new, functional tissue, as it facilitates cell–cell communication, extracellular matrix deposition, and uniform nutrient and oxygen diffusion throughout the scaffold.

**Fig. 5:**
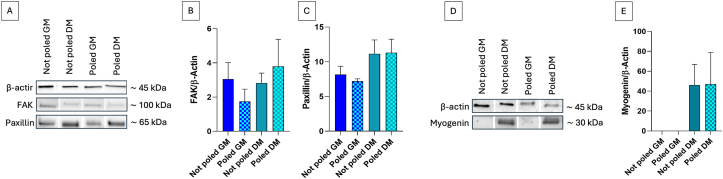
Western blot analysis. Representative cropped Western blot bands for FAK (A) and Paxillin (A) and Myogenin (D), along with the corresponding densitometric quantification (B) FAK, (C) Paxillin, and (E) Myogenin after 7 days of cell culture (*n* = 2). Protein expression levels are shown relative to β-actin, used as a control. No data are shown for the ‘Not poled GM’ and ‘Poled GM’ Myogenin groups, as no detectable bands were observed in the corresponding Western blot analyses; therefore, quantification was not possible. If not indicated, differences are not statistically significant.

To further investigate the mechanisms underlying cell adhesion on the EWP:BTO 1:1 scaffolds, an immunofluorescence analysis of focal adhesion-related proteins was performed, focusing on FAK and Paxillin. These proteins are key regulators of focal adhesion dynamics, linking integrin-mediated adhesion to actin cytoskeleton organization and thereby controlling myoblast attachment, migration, and differentiation [62,63]. In this study, immunofluorescent detection of FAK and Paxillin at day 7 revealed their clear presence in C2C12 cells cultured on both poled and not poled scaffolds, under both GM and DM conditions. However, due to scaffold autofluorescence, no qualitative differences in the distribution or intensity of FAK and Paxillin staining were observed among the groups (Fig. 4K and L). For this reason, we performed Western blot analysis to obtain a more reliable quantification of focal adhesion–related proteins. The results confirmed that both FAK and Paxillin were expressed in all experimental groups (Fig. 5A, B, C). Although no statistical difference was detected, a trend toward higher protein levels was observed in both poled and non-poled scaffolds in DM, particularly for Paxillin (Fig. 5C). This suggests that the EWP:BTO 1:1 scaffold, regardless of its polarisation, supports the formation and maturation of focal adhesion complexes. Increased expression of FAK and Paxillin is typically associated with enhanced cell–matrix adhesion, improved cytoskeletal organisation, and active mechanotransduction in cells cultured on biomaterial scaffolds, indicating that the material provides a favourable microenvironment for myoblast attachment and early differentiation. To specifically verify the ability to induce cell differentiation, the presence of Myogenin protein, a marker of early to mid-stage differentiation essential for proper skeletal muscle development and maturation, was investigated [64]. The data showed that Myogenin was produced only in DM condition in C2C12 cells seeded on both poled and not poled scaffolds, with no significant differences between them (Fig. 5D and E). This suggests that the scaffold supports the induction of the myogenic program independently of its polarisation status, providing a microenvironment permissive for myogenic differentiation.

Building upon the promising results obtained on cell proliferation and colonization on poled scaffolds compared to not poled ones, as well as the initial differentiation detected by Western blot, an additional set of experiments was designed to investigate whether the application of US stimulation could further modulate cellular responses. In detail, the aim was to evaluate whether the mechanical activation of the poled BTO particles through US could elicit electromechanical transduction within the scaffold, thereby enhancing cell adhesion, proliferation, and myogenic signalling. Specifically, after 3 days of culture on EWP:BTO 1:1 scaffolds, C2C12 cells were exposed to daily ultrasound stimulation (i.e. 60 s per day for 5 consecutive days, 900 mW/cm^2^) under both GM and DM conditions. As a preliminary investigation, cell viability and proliferation over time were assessed by the MTT assay. Ultrasound application began three days after cell seeding on the scaffolds. The results showed that US exposure did not negatively affect cell viability in any condition (Fig. 4A and B). After seven days of culture, corresponding to five US stimulations, a slight but consistent increase in metabolic activity was observed in the poled scaffolds, both in GM and DM conditions, suggesting that the local deformation of poled BTO particles induced by US may generate charges in their vicinity, which could modulate biological processes.

**Fig. 6:**
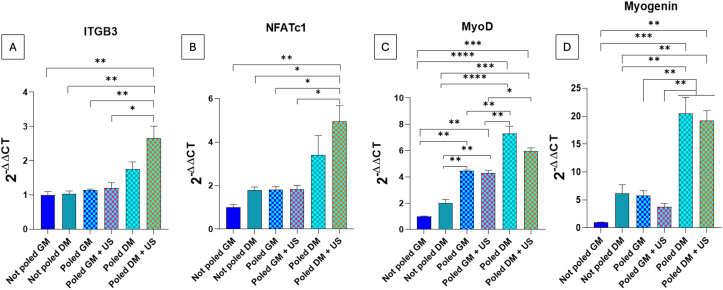
Relative quantification (2^−ΔΔCt^) of ITGβ3 (A), NFATc1 (B), MyoD (C), and Myogenin (D) gene expression after 7 days of culture under different conditions. Data are presented as mean ± SEM relative to the not poled GM group, used as the control (*n* = 4). ∗p value ≤ 0.05, ∗∗p value ≤ 0.01, ∗∗∗p value ≤ 0.001 and ∗∗∗∗p value ≤ 0.0001, where not indicated, differences are not statistically significant.

Based on these observations, a deeper investigation was performed to evaluate the molecular mechanisms underlying these effects. Gene expression analysis was conducted focusing on four target genes (i.e. ITGβ3, NFATc1, MyoD, and Myogenin) representing distinct yet interconnected pathways involved in cell adhesion, mechanotransduction, and myogenic differentiation. The expression of ITGβ3, the gene encoding β3-integrin, remained low and comparable in not poled scaffolds. In poled scaffolds, a modest increase was observed in GM, slightly enhanced by US stimulation (Fig. 6A). Notably, ITGβ3 was strongly upregulated in poled scaffolds under DM, and the combination of polarisation, DM, and US produced the highest expression, statistically significant. This pattern, in line with the physiological role of β3-integrin in initiating myogenesis in adult muscle and its upregulation during C2C12 myoblast differentiation [65], suggests that scaffold polarisation, combined with differentiation cues and US, synergistically promotes integrin-dependent adhesion and mechanosensitive signalling. Considering that β3-integrin is a focal adhesion protein, and that focal adhesions mediate cell migration necessary for cell fusion and myotube formation, this environment likely creates optimal conditions for myogenic progression.

NFATc1, a transcription factor responsive to Ca^2+^-dependent signalling, was also upregulated, showing a particularly significant increase in the poled + US group under differentiation conditions (Fig. 6B). Given the piezoelectric properties of the EWP:BTO scaffolds, these findings support the hypothesis that polarisation, together with US-induced mechano-electrical conversion, enhances intracellular Ca^2+^ influx and activates NFATc1-dependent transcriptional cascades contributing to myogenic progression. In fact, the NFATc1 pathway, a Ca^2+^/calcineurin-dependent signalling cascade, regulates gene expression involved in several cellular processes, including myoblast fusion and myogenic differentiation [66,67].

Finally, we focused on the myogenic regulators MyoD, constitutively expressed in many myogenic cell lines, and Myogenin, which is induced by differentiation cues and expressed during the early to mid-stages of myogenic differentiation [[68], [69], [70]]. Both genes were upregulated in all experimental groups compared to the not poled scaffold in GM, which served as the control (Fig. 6C and D). MyoD levels in cells on poled scaffolds in GM exceeded those in not poled scaffolds in DM, suggesting that scaffold polarisation can partially compensate for, or even surpass, the effect of differentiation medium, likely by promoting proliferation in line with cell proliferation data. An increased cell number may enhance fusion events and trigger the differentiation program, leading to elevated MyoD expression (Fig. 6C). Myogenin was consistently over-expressed across all conditions, with poled scaffolds showing the highest levels, highlighting their strong influence on transcriptional programs driving terminal differentiation (Fig. 6D). The absence of a clear synergistic effect of US stimulation on either gene suggests that the electroactive microenvironment provided by scaffold polarisation alone is sufficient to promote the initial myogenic progression.

Overall, gene expression analysis demonstrates that scaffold polarisation is the main driver enhancing cell adhesion, mechanotransduction, and myogenic signalling. The over-expression of ITGβ3 further confirms the above findings, supporting the evidence that scaffold polarisation promotes cell adhesion and colonization, likely through enhanced integrin-mediated signalling. A synergistic effect with US stimulation was particularly evident for ITGβ3 and NFATc1 genes, involved in early mechanosensitive and electroactive pathways, supporting the notion that US-induced piezoelectric activation can potentiate initial cell–material interactions and Ca^2+^-dependent signalling. At the analysed time point, the myogenic regulators MyoD and Myogenin were predominantly influenced by scaffold polarisation, with no appreciable enhancement from US stimulation. However, longer culture times and repeated US applications may further clarify whether mechano–electrical activation can also modulate later stages of myogenic differentiation.

### 3.6 *In vivo* biological evaluation

**Fig. 7:**
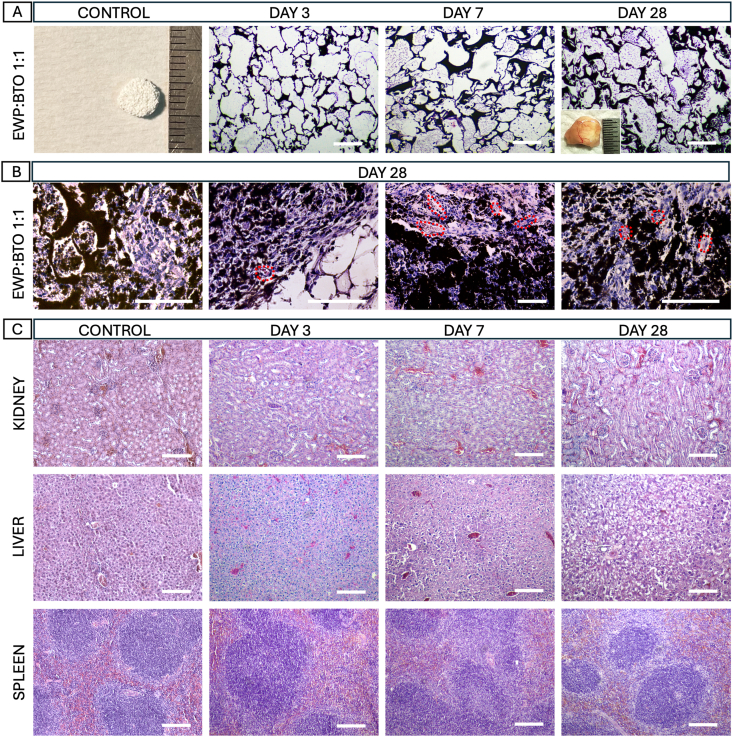
*In vivo* biological evaluation following subcutaneous implantation in mice (*n* = 3). **A**. EWP:BTO 1:1 sample prior to implantation and after explantation at 3, 7, and 28 days, stained with Haematoxylin and Eosin staining. **B**. Higher-magnification images of the 28-day explants. Red dashed circles indicate newly formed blood vessels. **C**. Organs (kidney, liver and spleen) explanted in control mice and after 3, 7 and 28 days of subcutaneous scaffold implantation. Scale bars: 100 μm.

To evaluate the *in vivo* biocompatibility of the EWP:BTO 1:1 scaffolds, a pilot study was conducted by subcutaneously implanting the scaffolds in mice. Macroscopic observations performed at 3, 7, and 28 days post-implantation revealed that the scaffolds remained in place, with no signs of inflammation, infection, or tissue toxicity at any time point. The implanted scaffolds maintained their structural integrity throughout the study period, consistent with their *in vitro* stability profile (Fig. 7A). Moreover, a progressive increase in host cell–deposited extracellular matrix within the scaffold pores was observed over time. Notably, by day 28, many pores appeared completely filled, and clear evidence of neovascularization was detected both around the scaffolds and within their pores, indicating good biocompatibility and host tissue integration (Fig. 7B). Histological analysis of the harvested organs—including kidney, liver, and spleen—showed no systemic toxicity (Fig. 7C). No signs of local or systemic adverse effects were observed, indicating that the EWP:BTO 1:1 scaffolds are well tolerated *in vivo*. These findings support the potential of the EWP:BTO 1:1 scaffold for further preclinical applications. In particular, future studies could explore orthotopic implantation in muscle tissue to better replicate the native environment and assess tissue regeneration under physiologically relevant conditions. Additionally, the combination of scaffold implantation with ultrasound-mediated piezoelectric activation could be investigated to enhance cellular response and functional tissue formation, leveraging the scaffold's piezoelectric properties to stimulate muscle regeneration *in situ*.

## 4 Conclusion

In this study, we developed and characterised novel piezoelectric scaffolds based on egg white proteins (EWP) integrated with barium titanate (BTO) particles, using a rapid and versatile microwave-assisted fabrication method, aiming to establish a bioactive platform for wireless, ultrasound-driven electrical stimulation in skeletal muscle regeneration. The optimized EWP:BTO 1:1 composition demonstrated the most favourable combination of porosity, degradation stability, and mechanical compliance, closely mimicking native muscle tissue properties. Corona poling successfully induced a macroscopic polarisation of the embedded BTO phase, enabling electromechanical responsiveness under ultrasound stimulation.

Combined physicochemical characterization, *in vitro* biological evaluation, and a pilot *in vivo* assessment validate EWP:BTO piezoelectric scaffolds as a sustainable, biocompatible, and functionally active platform. Notably, ultrasound stimulation synergized with scaffold polarisation to enhance early mechanosensitive and electroactive responses. By coupling structural biomimicry with dynamic electromechanical cues, this system represents an early-stage step toward the development of non-invasive, 4D regenerative strategies for VML, supporting its suitability for further functional evaluation in orthotopic muscle models under controlled ultrasound activation.

## Ethics approval and consent to participate

All experimental *in vitro* procedures involved commercially available murine cell line. Animal experimentation was carried out at i3S - Instituto de Investigação e Inovação em Saúde animal facility, approved by the local Animal Ethics Committee and by Direcção Geral de Alimentação e Veterinária through licence n° 122395/24-S 22-10-2024 and conducted in accordance with the European Legislation on Animal Experimentation through Directive 2010/63/UE.

## CRediT authorship contribution statement

**Noemi Ravaglia:** Formal analysis, Investigation, Methodology, Writing – original draft, Writing – review & editing. **Arianna Rossi:** Formal analysis, Investigation. **Maurizio Vignolo:** Formal analysis, Investigation, Methodology, Writing – original draft. **Pietro Galizia:** Formal analysis, Methodology, Writing – review & editing. **Diana Pacheco:** Formal analysis, Investigation, Methodology, Writing – original draft. **Federica Arienti:** Methodology. **Carlo Baldisserri:** Investigation, Methodology, Writing – review & editing. **Caitlin M. Guzzo:** Investigation, Methodology. **Monica Montesi:** Writing – original draft, Writing – review & editing. **Rosa Mancinelli:** Writing – review & editing. **Massimiliano Labardi:** Formal analysis, Investigation, Visualization, Writing – original draft, Writing – review & editing. **Tatiana M.F. Patrício:** Formal analysis, Investigation. **Julia Glaum:** Funding acquisition, Investigation, Writing – original draft, Writing – review & editing. **Carla Cunha:** Formal analysis, Investigation, Methodology, Writing – original draft. **Elisa Mercadelli:** Formal analysis, Writing – review & editing. **Giorgio Luciano:** Conceptualization, Formal analysis, Investigation, Methodology, Writing – original draft. **Silvia Panseri:** Conceptualization, Funding acquisition, Supervision, Writing – original draft, Writing – review & editing.

## Declaration of competing interest

The authors declare no conflict of interest.
