# Supplementary material for: 4D piezoceramic-integrated scaffolds with bioelectric cues for skeletal muscle regeneration
Source: Bioact Mater. 2026 Jun 24;65:1005–18. doi: 10.1016/j.bioactmat.2026.06.027 (PMC13320252; doi:10.1016/j.bioactmat.2026.06.027)
Supplement: Multimedia component 1 [file mmc1.docx]

4D Piezoceramic-Integrated Scaffolds with Bioelectric Cues for Skeletal Muscle Regeneration

**SUPPORTING INFORMATION**

*1. Piezoelectric scaffolds synthesis*


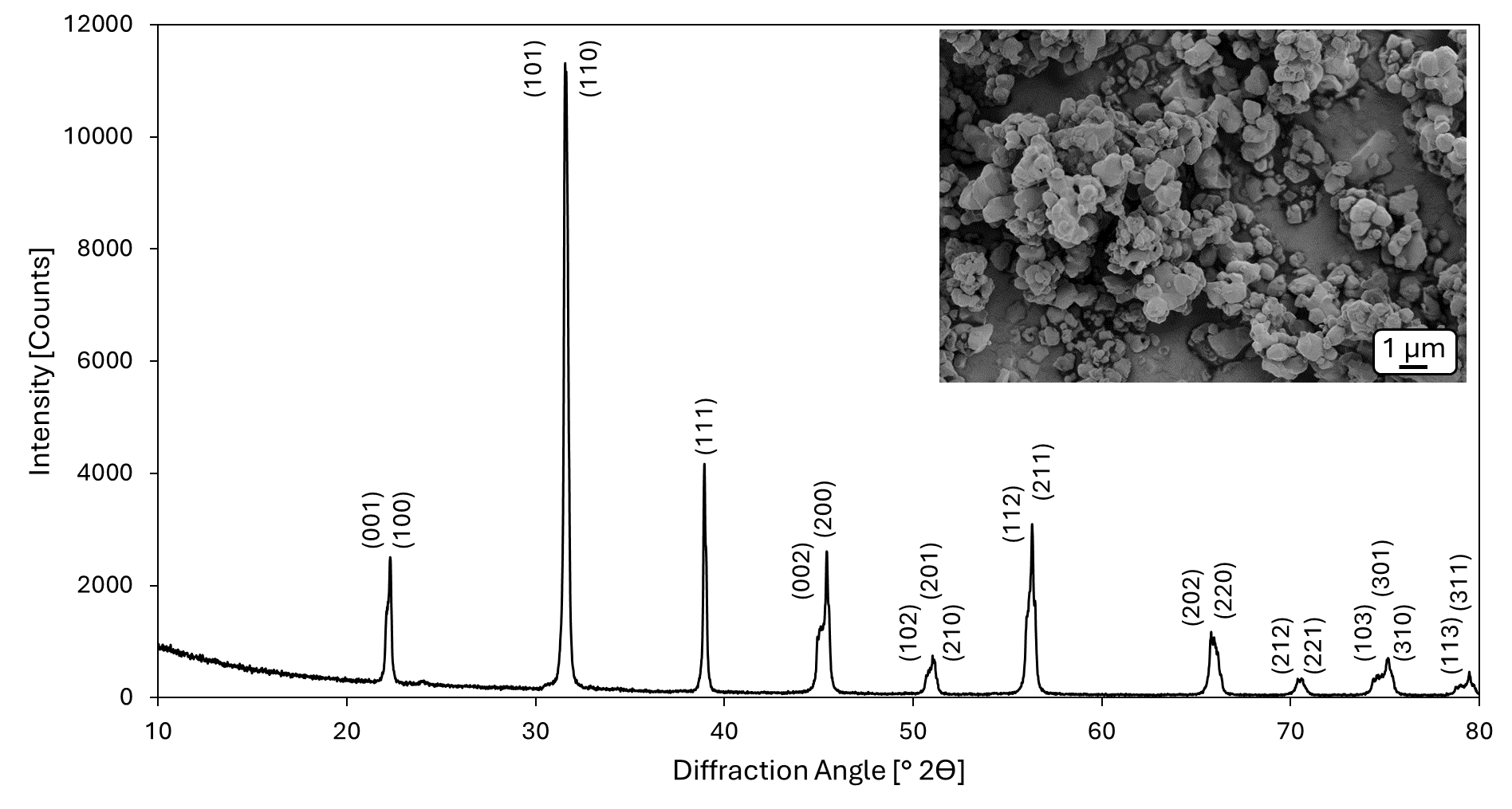
**Figure SI 1**. X-ray crystallography pattern of BTO powders (Reference PDF # 81-2203). The inset shows SEM micrograph of the BTO powder used as starting piezoelectric active phase.

*2. Transverse piezoresponse of scaffolds*

*2.1 Broadband dielectric spectroscopy of pressed pellets*

Pressed pellets were obtained from the powder obtained by crushed scaffolds by a uniaxial press, by applying 1 ton/cm^2^ pressure for 30 sec. 13 mm diameter pellets were obtained. Thickness, weight, and corresponding density of each pellet are reported in Table SI 1.

Table SI 1. Thickness, weight, and corresponding density values of the produced pressed pellets

| Sample (pellet) | Thickness (mm) | Weight (mg) | Density (kg/m^3^) |
| --- | --- | --- | --- |
| EWP:BTO 1:0 Not Poled | 300 | 29.5 | 740 |
| EWP:BTO 1:0 Poled | 422 | 41.9 | 750 |
| EWP:BTO 1:1 Not Poled | 932 | 301 | 2430 |
| EWP:BTO 1:1 Poled | 1430 | 475 | 2500 |

The average density value of the two EWP:BTO 1:0 pellets (745 kg/m^3^) will be assumed as the density of the egg white proteins at ambient conditions (*T* = 25°C, *RH* = 50%).

Broadband Dielectric Spectroscopy (BDS) was performed with an Alpha Analyzer spectrometer by Novocontrol technologies GmbH & Co. (Montabaur, Germany), equipped with a Novocontrol Quatro nitrogen gas flow cryostat. The dielectric measurements were achieved on the pellets placed between two parallel electrodes in the active cell. Measurements were performed under a sinusoidal applied voltage of 1.0 V. Impedance spectra were obtained by recording the resulting current while ramping the frequency *f* of the applied voltage in the range from 10^−1^ Hz to 10^6^ Hz, with logarithmic increments, at room temperature. The measurement time was about 5 min. Two series of measurements were performed. The first were made on as-produced pellets, indicated as measurements in air. The second were made after keeping the pellets under moderate vacuum (10^-3^ mBar) for 4 days, indicated as measurements in vacuum-N_2_, and after transfer into the BDS measurement chamber, dry nitrogen atmosphere was maintained. Measurements were repeated twice, always resulting as coincident, showing that water uptake during transfer was negligible.

Analysis of dielectric relaxation processes occurring in the protein samples, in the cases of high (air samples) and low (vacuum-N_2_ samples) hydration, have be conducted by fitting of dielectric spectra, by a methodology already demonstrated to be effective on polysaccharide films [1]. In essence, dielectric loss tangent (tan *δ*) spectra are fitted by a combination of Havriliak-Negami functions describing primary (α), secondary (β), Maxwell-Wagner-Sillars (interfacial) and electrode polarizations, and the conductivity contribution represented by an inverse-power with a non-integer exponent. This procedure is immune to thickness variations of samples due to mechanical relaxation or temperature changes.

*2.2. Piezoresponse force microscopy of single particles.*

Converse piezoelectric coefficient *d*_33_ of single BTO particles was measured by Piezoresponse Force Microscopy (PFM). A recently introduced non-contact technique [2] has been used that allows measurement of particles adhering to a flat, conducting substrate avoiding dragging of the sample during scanning. An atomic force microscope (AFM) was adapted to the non-contact PFM method. The AFM used was a NanoScope IIIa with MultiMode head, equipped with gas cell and ADC5 extension (Veeco Instruments Inc., USA). Non-contact type AFM cantilevers (Nanosensors PPP-NCLPt, platinum-iridium coated silicon tips, spring constant ~ 40 N/m, resonant frequency *f*_0_ ~ 156 kHz, quality factor *Q*_0_ ~ 500 in air, tip radius ~ 30 nm) were operated in constant-excitation frequency modulation (CE-FM)-AFM mode, with a free oscillation amplitude of *A*_0_ ~ 20 nm. Distance stabilization is obtained by feedback on the oscillation amplitude. An oscillating voltage *V*(*t*) = *V*_dc_ + *V*_ac_ cos(2π*ft*) is applied to the probe as customary in PFM, with the sample conductive substrate grounded. The oscillation amplitude signal *A_f_*(*t*) from the FM-AFM controller (PLLProII, RHK Technology, Troy, USA) is demodulated at frequency *f* by a dual lock-in amplifier (SRS830DSP, Stanford Research Systems, USA) whose output (Δ*A*) was acquired through the auxiliary acquisition channels of our AFM. The latter output represents the measurement of the surface displacement induced by the applied electric field, and therefore of the converse piezoelectric effect. By dividing such surface displacement by the applied voltage, the piezoelectric coefficient *d*_33_ is obtained. Particles were deposited on a doped silicon substrate by spin coating of a diluted dispersion (0.5 mg/mL) in acetone, with no previous sonication. Spin coating parameters were: acceleration 7000 rpm/s, speed 7000 rpm, spinning time 30 s.

*2.3. Broadband dielectric spectroscopy of pellets*

Dielectric spectra of the produced pellets are shown in Figure SI 1. Poled samples exhibit higher dielectric constant, of about 20-30%, in all cases. The much higher dielectric constant measured in ambient conditions is likely due to ionic conductivity occurring through absorbed water molecules. Such conductivity enhances polarization phenomena at low frequency, such as Maxwell-Wagner-Sillars (MWS), or interfacial, polarization, due to charge accumulation and transport at interfaces inside the material, as well as electrode polarization, that is a measurement effect due to the BDS electrodes contacting the pellets [3]. In dry conditions, such effects become more moderate, at least in the PiezoGauge frequency range (100-300 Hz). Values of the dielectric constant at the frequency of 222 Hz for all cases are reported in Table SI 2. These values were used for the determination of the transverse piezoelectric coefficients by PiezoGauge.


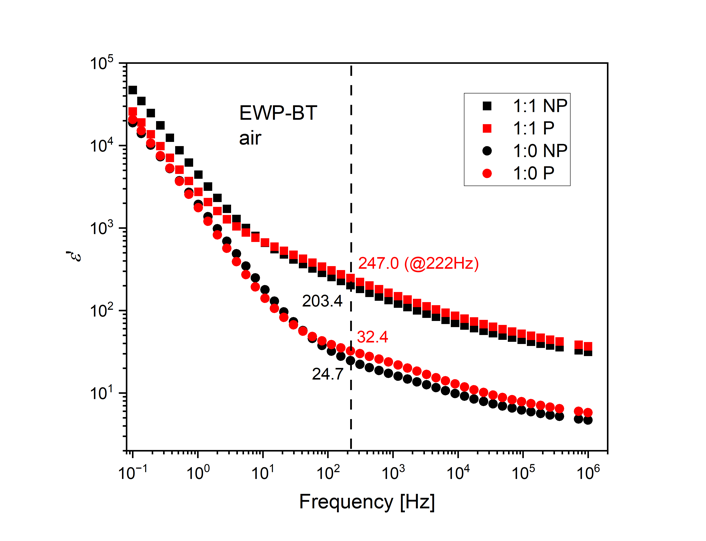
(a)
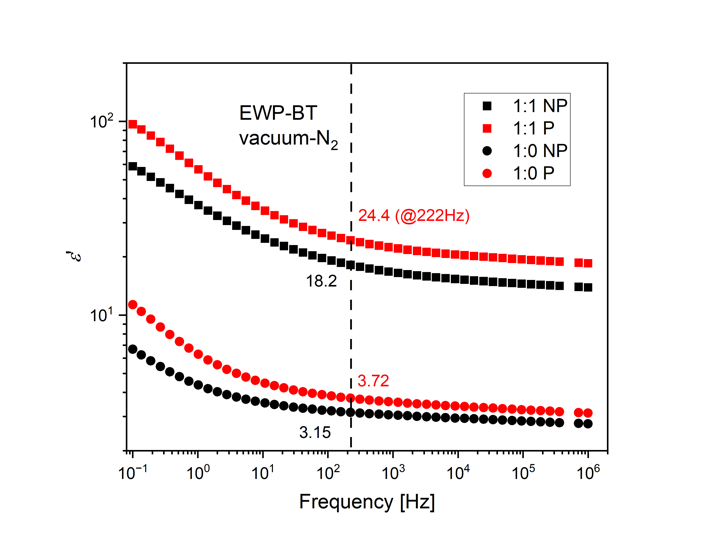
(b)

Figure SI 2. BDS dielectric constant spectra of the produced pressed pellets in air (a) as well as dry conditions (b), in not poled (NP) as well as in poled (P) cases.

Table SI 2. Dielectric constant values of the produced pressed pellets at a typical frequency value of PiezoGauge measurement, in ambient and dry conditions. Measurements were conducted at *T* = 25°C unless specified.

| Sample (pellet) | *ε*' @ 222 Hz  (air) | *ε* ' @ 222 Hz  (vacuum-N_2_) | *ε* _∞_  (air) | *ε* _∞_  (vacuum-N_2_) |
| --- | --- | --- | --- | --- |
| EWP:BTO 1:0 Not poled | 24.7 | 3.15 | 2.91 (from fit) | 2.51 (from fit) |
| EWP:BTO 1:0 Poled | 32.4 | 3.72 |  | 2.66 (*T* = -140°C) |
| EWP:BTO 1:1 Not poled | 203.4 | 18.2 |  |  |
| EWP:BTO 1:1 Poled | 247.0 | 24.4 |  |  |

Dielectric analysis for the EWP:BTO 1:0 not poled pellet in air conditions is shown in Figure SI 2(a). The dielectric signal at low frequency is dominated by conductivity contributions, however the primary (α) and secondary (β) relaxations of the protein can be detected, with relaxation frequency at room temperature of 3.41 kHz and 54.3 kHz, respectively. In dry conditions, the primary process is not detectable, while the secondary process shows higher relaxation frequency (147.9 kHz). Slowing down of relaxation processes in air-exposed samples is expected because of a possible plasticization effect of the protein by absorbed water.

*
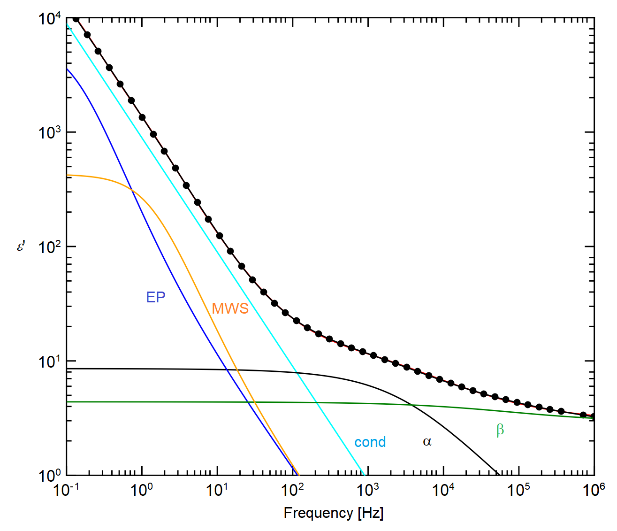
* (a) *
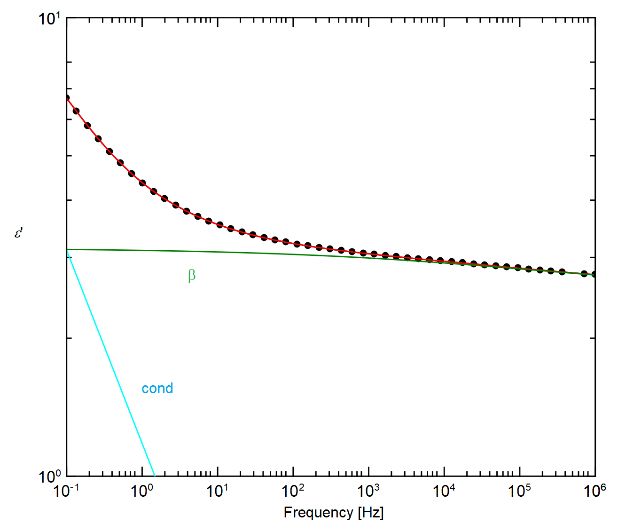
* (b)

Figure SI 3. Fitting analysis of the dielectric data for the EWP:BTO 1:0 NP pellet, in the air (a) and vacuum-N_2_ (b) cases. Contributions of conductivity effects (indicated by labels cond, EP, MWS in figure) are much larger in the air case, due to the abundance of hydration water.

*2.4. Piezoresponse of single BTO particles*

In Figure SI 4 the topographic AFM image of a typical particle cluster deposited on doped silicon is shown. Particle size of around 400 nm was estimated from the cluster height.

**Figure SI 4**. AFM topography of a BTO particle cluster deposited on doped silicon (image size 2 μm x 2 μm).

In Figure SI 5, zooming on a particle portion is shown, with simultaneous topography (a) and piezoresponse (b) imaging. Line profiles corresponding to the position indicated on such images are shown in (c). Reliable piezoresponse values are those corresponding to the particle top and clean substrate, as highlight in the graph. The remaining values concern contaminated substrate portions or the particle shank and should therefore be excluded from the analysis.

 (a)

 (b)


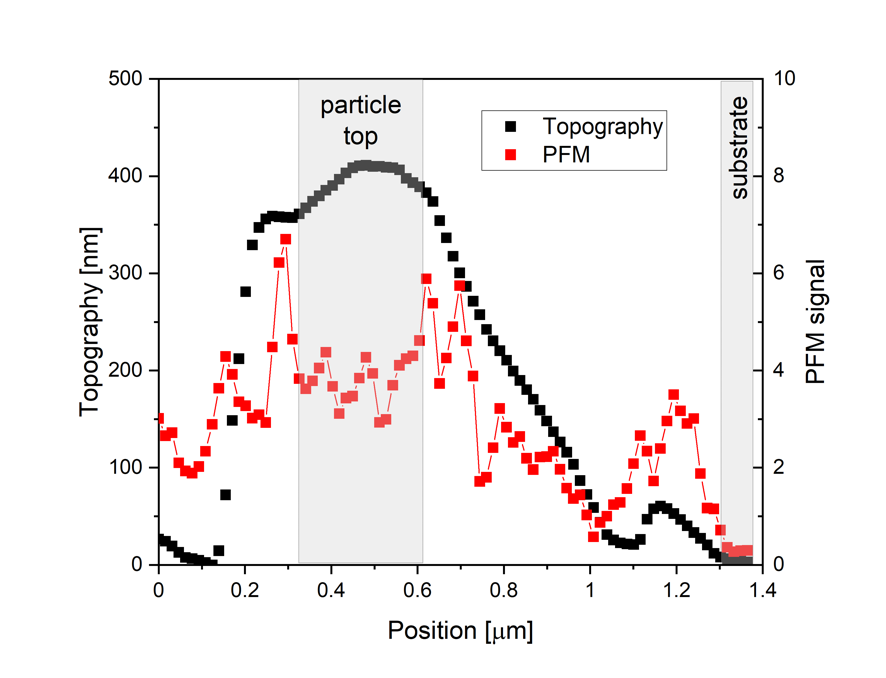
 (c)

**Figura SI 5.** (a) Topography (image lateral size 1.4 μm) and (b) corresponding piezoresponse image (PFM). (c) Line profiles at the positions marked on (a) and (b), with the indications of the reliable regions for the analysis, i.e., particle top and clean substrate.

By locating the AFM probe on the reliable regions, the piezoresponse signal was recorded at variance of the applied *ac* potential, *V*_ac_ (Figure SI 6). Values up to 34 ± 3 pm/V were measured on the particles, not affected by electrostatic artifacts, as documented by the corresponding measurement on the substrate, yielding a maximum value of the electrostatic contribution of 4.2 pm/V. The lock-in detector used for piezoresponse measurements provides in the latter case a phase-locked signal with opposite phase than the one on the particles, therefore the electrostatic should contribute to underestimate the particle response. The deviation from a linear trend observed for the particle can be due to drifts of the probe position during the measurement time. Indeed, the piezoresponse recorded on particles is not homogeneous, as also visible from the piezoresponse map of Figure SI 4 (b). Notably, a phase-locked signal is detected on the particle also for the lowest value of the applied potential (0.1 V), while this is not the case on the substrate for the same potential value.


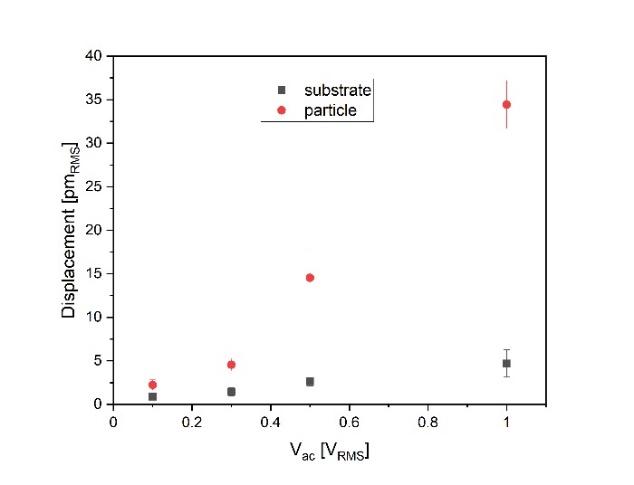


**Figure SI 6.** PFM piezoresponse on the particle as well as on the substrate, for different values of the applied potential.


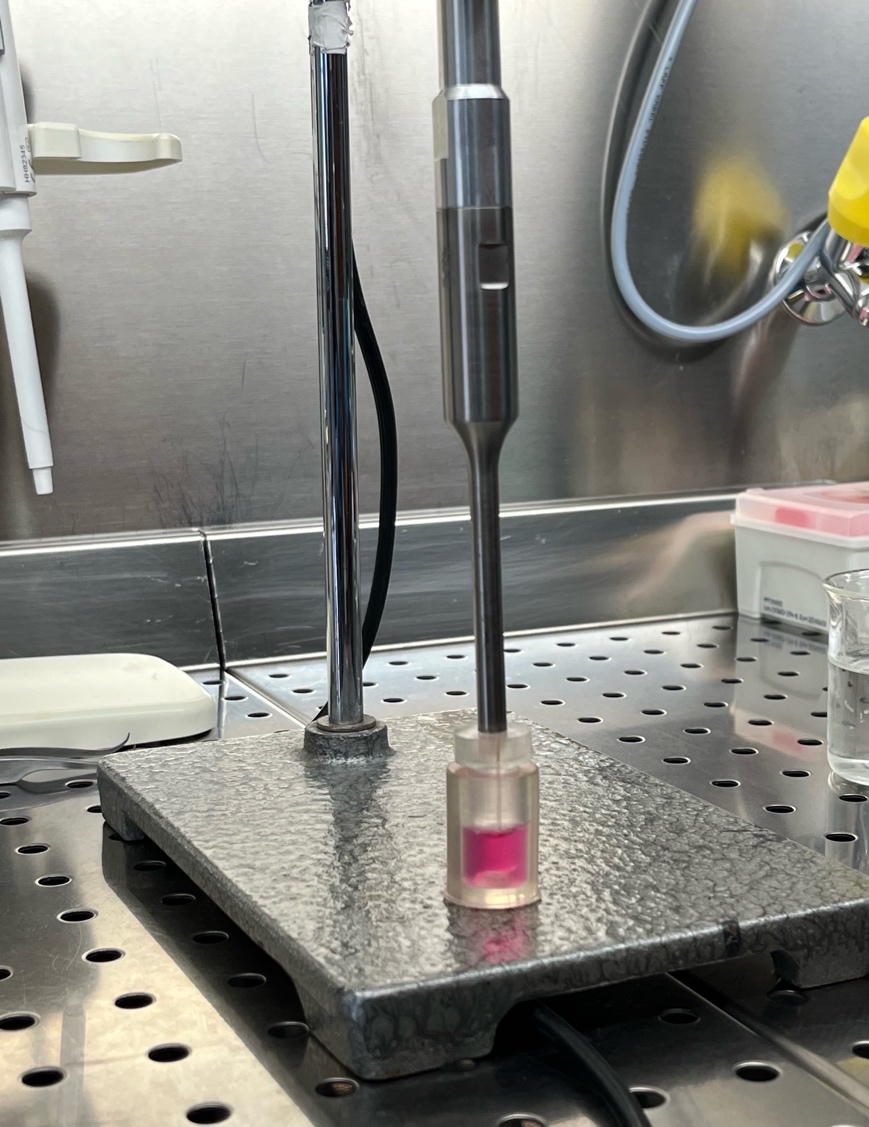
3. Ultrasound-mediated piezoelectric stimulation

US stimulations were carried out operating the Vibra-Cell ultrasonic processor at 26W power intensity. Based on calculations derived from the data [4], a 2-cm-thick layer of 10:1 cross-linked PDMS (thickness of the PDMS cap) is expected to reduce ultrasound power transmission by approximately two orders of magnitude, in order to obtain the final stimulation intensity used of 900 mW/cm². The PDMS chamber and cap (Fig. SI 7) were made according to the following procedure: the base component was mixed with the curing agent (base to curing ratio 10:1) to start the addition crosslinking polymerization reaction. The mixture was then poured into a custom-made mould and left overnight at room temperature to obtain the silicone reticulation. Furthermore, a PDMS lid with a hole in the centre was fabricated to have the best fitting between the US probe and the PDMS culture chamber.

**Figure SI 7** PDMS chamber


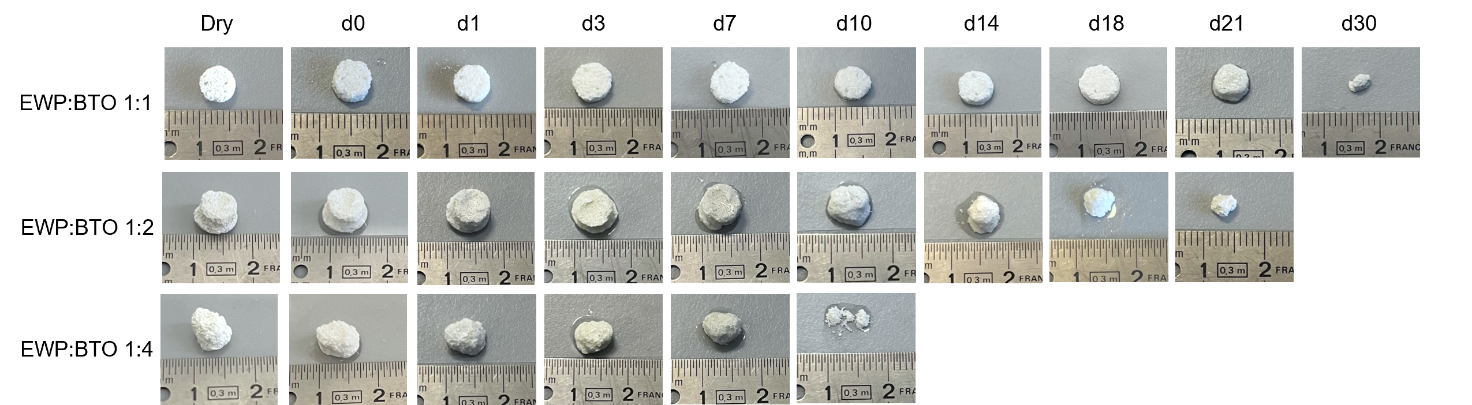


**Figure SI 8. Scaffold degradation analysis.** Representative images of the scaffolds collected at different time points during the degradation test in PBS at 37 °C.

**Video SI 1.** 3D micro-CT reconstruction of the sample EWP:BTO 1:1

**Video SI 2.** 3D micro-CT reconstruction of the sample EWP:BTO 1:2

**Video SI 3.** 3D micro-CT reconstruction of the sample EWP:BTO 1:4

**SI References**

1. M. Labardi, M. Montorsi, S. Papa, L.M. Ferrari, F. Greco, G. Scarioni, S. Capaccioli, Glass transition and crystallization of chitosan investigated by broadband dielectric spectroscopy, Polymers 17 (2025) 2758. https://doi.org/10.3390/polym17202758.

2. M. Labardi, A. Magnani, S. Capaccioli, Piezoelectric displacement mapping of compliant surfaces by constant-excitation frequency-modulation piezoresponse force microscopy, Nanotechnol 31 (2020) 075707. https://doi.org/10.1088/1361-6528/ab52ca.

3. F. Kremer, A. Schoenhals, Eds., Broadband Dielectric Spectroscopy, Springer-Verlag GmbH: Berlin, Heidelberg, Germany (2003).

4. A. Cafarelli, A. Verbeni, A. Poliziani, P. Dario, A. Menciassi, L. Ricotti, Tuning acoustic and mechanical properties of materials for ultrasound phantoms and smart substrates for cell cultures, Acta Biomater 49 (2017) 368–378. https://doi.org/10.1016/j.actbio.2016.11.049.
